# Suicide in Adolescents with Mood Disorders

**DOI:** 10.1192/j.eurpsy.2022.381

**Published:** 2022-09-01

**Authors:** K. Shah, C. Trivedi, D. Kamrai, S. Srinivas, Z. Mansuri

**Affiliations:** 1 Griffin Memorial Hospital, Psychiatry, Norman, United States of America; 2 Texas Tech University Health Science Center at Permian Basin, Psychiatry, Midland, United States of America; 3 A.J. Institute of Medical Sciences and Research Center, Psychiatry, Manglore, India; 4 Boston Childrens Hospital/Harvard Medical School, Psychiatry, Brighton, United States of America

**Keywords:** Suicide, Adolescents, DMDD, Mood disorders

## Abstract

**Introduction:**

Adolescents patients presenting with mood disorders, including disruptive mood dysregulation disorder (DMDD), often present with the comorbid disorders such as oppositional defiant disorder (ODD) and attention-deficit hyperactivity disorder (ADHD).

**Objectives:**

1) Evaluate the association between suicide in adolescents and various mood disorders. 2) To study the impact of comorbid conditions in DMDD on suicide ideation and attempt in adolescents.

**Methods:**

We used 2016-2017 National Inpatient Sample dataset to select patients with mood disorders. Rao Scott adjusted Chi-Square test used to compare the groups with SPSS v26.

**Results:**

In this study, 15195 patients were in the DMDD group (Mean age:12.1,F: 38%) and 219205 in the ‘other mood disorders’ group (Mean age:14.4,F:67%). The odds of SI/SA were two times more in patients with the ‘other type of mood disorder’ (OR:2.07, 95%CI: 1.77-2.14). Patients with the primary diagnosis of DMDD sub-classified into four groups (Group 1: DMDD only (n=5160), Group 2: DMDD+ADHD (n=7240), Group 3: DMDD +ODD (n=700), and Group 4: DMDD+ADHD+ODD (n=2095). SI/SA was prevalent in 30.8%, 26.0%, 22.9% and 26.3% in Group 1, 2, 3 and 4 respectively (p: 0.03). SI/SA was more prevalent in females compared to males (31.3% vs. 25.2%). An increase of 1 year in age was associated with a higher SI/SA (OR:1.05, 95%CI:1.01-1.08, 0.01). The SI/SA odds were 5% more in female patients (OR:1.27, p:0.01).

**Conclusions:**

The study reveals that the risk of suicide ideation or suicide attempt is almost twice in the adolescent with mood disorders without DMDD compared to the DMDD group.

**Disclosure:**

No significant relationships.

